# Three-dimensional structure of recombinant type 1 inositol 1,4,5-trisphosphate receptor

**DOI:** 10.1042/BJ20100143

**Published:** 2010-05-27

**Authors:** Francis Wolfram, Edward Morris, Colin W. Taylor

**Affiliations:** *Department of Pharmacology, University of Cambridge, Tennis Court Road, Cambridge CB2 1PD, U.K.; †Section of Structural Biology, Institute of Cancer Research, Chester Beatty Laboratories, London SW3 6JB, U.K.

**Keywords:** calcium channel, electron microscopy (EM), inositol 1,4,5-trisphosphate receptor (IP_3_R), single-particle analysis (SPA), CLM, cytosol-like medium, DDM, dodecyl maltoside, ECFP, enhanced cyan fluorescent protein, EM, electron microscopy, ER, endoplasmic reticulum, IP_3_R, inositol 1,4,5-trisphosphate receptor, PBM, phosphate-buffered medium, PEG, poly(ethylene glycol), RyR, ryanodine receptor, SPA, single-particle analysis, TEM, Tris/EDTA medium

## Abstract

IP_3_Rs (inositol 1,4,5-trisphosphate receptors) are the intracellular channels that mediate release of Ca^2+^ from the endoplasmic reticulum in response to the many stimuli that evoke Ins(1,4,5)*P*_3_ formation. We characterized and purified type 1 IP_3_R heterologously expressed in Sf9 insect cells, and used the purified IP_3_R1 to determine its three-dimensional structure by electron microscopy and single-particle analysis. Recombinant IP_3_R1 has 4-fold symmetry with overall dimensions of approx. 19.5 nm×19.5 nm×17.5 nm. It comprises a small domain, which is likely to include the pore, linked by slender bridges to a large cytoplasmic domain with four petal-like regions. Our structures of recombinant IP_3_R1 and native cerebellar IP_3_R have similar appearances and dimensions. The only notable difference is the absence of a central stigma-like domain from the cytoplasmic region of recombinant IP_3_R1. The first structure of a recombinant IP_3_R is an important step towards developing three-dimensional structures of IP_3_R that better contribute to our understanding of the structural basis of IP_3_R activation.

## INTRODUCTION

Cytosolic Ca^2+^ signals regulate diverse cellular activities and most of these signals arise from regulated opening of Ca^2+^-permeable channels. IP_3_Rs (inositol 1,4,5-trisphosphate receptors) are the most widely expressed of these channels [1]. Most IP_3_Rs are expressed in membranes of the ER (endoplasmic reticulum), but they are present also in the plasma membrane of some cells [2], in the nuclear envelope [3], in the Golgi apparatus [4] and perhaps also in secretory vesicles [5]. Three vertebrate genes encode closely related subunits of IP_3_Rs, each comprising ~2700 amino acid residues. The functional IP_3_R is a homo- or hetero-tetrameric assembly of these subunits [6–8]. Each subunit has a single Ins(1,4,5)*P*_3_-binding site, the Ins(1,4,5)*P*_3_-binding core (residues 224–604), which comprises two domains forming a clam-like structure that encloses a positively charged pocket to which Ins(1,4,5)*P*_3_ binds [9]. The N-terminal of the IP_3_R (residues 1–223), the suppressor domain, is the only other region of the IP_3_R for which a high-resolution structure is available [10]. The head of its hammer-like structure forms a β-trefoil, whereas the handle comprises a helix–turn–helix motif [10]. Although the structural basis of IP_3_R activation remains poorly understood, it is clear that the suppressor domain provides an essential link between Ins(1,4,5)*P*_3_ binding to the Ins(1,4,5)*P*_3_-binding core and opening of the pore [11,12]. Furthermore, the N-terminal of RyRs (ryanodine receptors), another major family of intracellular Ca^2+^ channels, has a structure almost indistinguishable from that of the suppressor domain of IP_3_R1 [13], suggesting that both families of intracellular Ca^2+^ channels may share similar gating mechanisms. For both RyRs and IP_3_Rs, the Ca^2+^-permeable pore of the channel is formed by the last pair of the six transmembrane helices of each subunit together with the luminal loop that links them. Intermediate-resolution structures of RyR, derived from SPA (single-particle analysis) [14,15], together with sequence alignments [16,17] and mutagenesis [2,18] of both RyR [16,17] and IP_3_R [2,18,19] are consistent with the idea that the pore of both channels has a structure broadly similar to that of K^+^ channels [20], with each pair of transmembrane helices cradling an intervening pore helix and selectivity filter. A direct interaction between the suppressor domain and the cytosolic helix that links the fourth and fifth transmembrane domains may mediate gating of IP_3_R [11,12].

High-resolution structures of the complete IP_3_R are ultimately required if the structural basis of its activation is to be fully resolved. To date, five intermediate-resolution (~30 Å, where 1 Å=0.1 nm) structures of IP_3_R1 purified from cerebellum have been determined using EM (electron microscopy) and SPA [21–25]. These structures differ in their details, but they all show a 4-fold symmetry and have two distinct regions: a large cytoplasmic domain and a small domain, which is assumed to include the channel region [26]. The first three-dimensional structure to be published had the shape of an uneven dumbbell with a height of 17 nm, and with four arms radiating from the large cytoplasmic domain [22]. We presented a structure reminiscent of a flower, in which the stalk represents the channel domain, and the cytoplasmic regions are represented by the petals and stigma with overall dimensions of ~18 nm×18 nm×18 nm [23]. Serysheva et al. [24] described the structure of IP_3_R as a large cytoplasmic pinwheel with a smaller square-shaped transmembrane domain; its overall dimensions were ~25 nm×25 nm×19 nm. Hamada and Mikoshiba [28] compared the three-dimensional structures with and without Ca^2+^ and suggested that Ca^2+^ caused IP_3_R to switch from a mushroom-like (~19 nm×19 nm×16 nm) to a windmilllike (~22 nm×22 nm×18 nm) shape, with the head of the mushroom and the wings of the windmill representing cytosolic domains [21]. The most recent structure resembles a heavily fenestrated hot-air balloon with dimensions of ~17 nm×17 nm×23 nm [25]. In the 7–8 years since the publication of the first three-dimensional structures of native IP_3_R [22–24,28], there has been no significant progress towards the higher-resolution structures that are required if the structures are to contribute further to our understanding of the workings of IP_3_R.

One approach, with considerable potential to accelerate progress, is to extend EM and SPA analyses of the three-dimensional structure from native IP_3_R to recombinant proteins. The benefits might include simplified purification procedures (using tagged IP_3_R), isolation of more homogeneous protein samples than are likely to be present in native tissues, and the opportunity to introduce tags identifiable by EM to allow mapping of primary sequence to the three-dimensional structure. Similar approaches applied to RyR [29–32] have allowed our understanding of its three-dimensional structure to progress well beyond that of IP_3_R [33]. In the present study, we used EM and SPA to establish the first three-dimensional structure of recombinant IP_3_R1 heterologously expressed in Sf9 insect cells.

## EXPERIMENTAL

### Materials

Fetal bovine serum, primers, fungizone, gentamicin and BSA were from Sigma. ATP and Complete™ protease inhibitor cocktail were from Roche. DDM (dodecyl maltoside) was from Calbiochem. Restriction enzymes and most molecular biology reagents were from New England Biolabs. [^3^H]Ins(1,4,5)*P*_3_ (18 Ci/mmol) was from PerkinElmer Life Sciences, and Ins(1,4,5)*P*_3_ was from Alexis Biochemicals. PreScission protease and the HiTrap Q FF and Superose 6 10/300 GL columns were from GE Healthcare. Mag-fluo-4AM and Sf-900 II SFM medium were from Invitrogen. Other materials were from Sigma, Fisher Scientific or the sources specified in the text or earlier publications [12,34].

### Expression of IP_3_R1 in Sf9 cells

**Figure 1:**
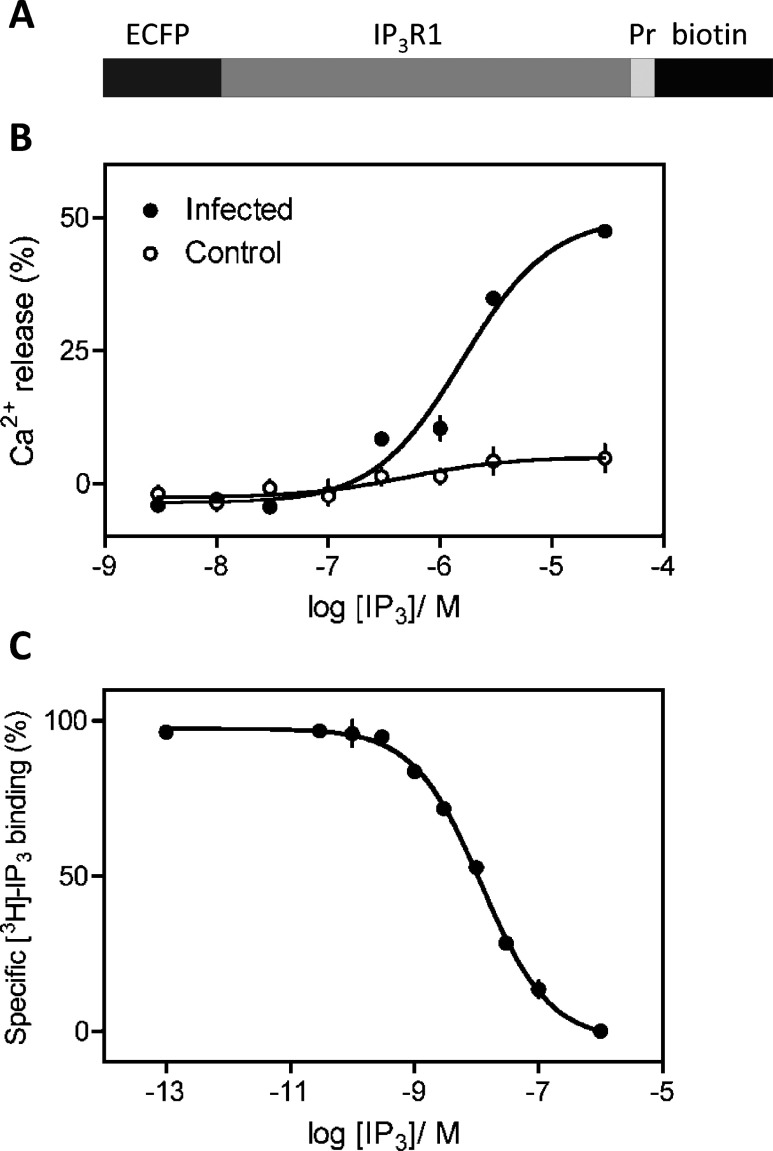
Recombinant IP_3_R1 is functional (**A**) The recombinant IP_3_R1 used has an N-terminal ECFP tag, a PreScission protease cleavage site (Pr) and the biotinylation sequence (biotin). (**B**) Ins(1,4,5)*P*_3_-evoked Ca^2+^ release from permeabilized Sf9 cells with or without expression of recombinant IP_3_R1. (**C**) Specific binding of [^3^H]Ins(1,4,5)*P*_3_ (1.5 nM) in the presence of the indicated concentrations of Ins(1,4,5)*P*_3_ (IP_3_) to membranes prepared from Sf9 cells expressing recombinant IP_3_R1. Results (**B** and **C**) are means±S.E.M (*n*=3).

A pENTR1A vector encoding IP_3_R1 N-terminally tagged with ECFP (enhanced cyan fluorescent protein) (ECFP–IP_3_R1) was prepared from two existing pENTR1A vectors containing full-length rat IP_3_R1 (GenBank® accession number GQ233032.1) and another containing an ECFP-tagged N-terminus of IP_3_R1 (residues 1–604) by ligation after digestion with NheI and KpnI. The product provided the template from which PCR was used to engineer a C-terminal biotinylation sequence and PreScission cleavage site (Figure 1A). The reverse primer (5′-TGCATCTCGAGTTATTCGTGCCATTCTATTTTTTGTGCTTCAAAGATGTCGTTGAGTCCGGGCCCCTGGAACAGAACTTCCAGGGCTGGCTGCTGTGGGTTGACATTCATGTG-3′) introduced the PreScission cleavage site and biotinylation sequence (Figure 1A). The forward primer [5′-ATGCAGAATTCGGATCCTGTACAACATGAGGCCCAAG-3′, corresponding to bases (underlined) 6326–6351 of IP_3_R1] used a unique BstBI site near the 3′ end of the open reading frame. The PCR product and ECFP–IP_3_R1 plasmid were digested with BstBI and XhoI and ligated to give a pENTR1A plasmid encoding the tagged IP_3_R1 shown in Figure 1(A). This IP_3_R1 construct was then transferred into artificial baculovirus DNA (BaculoDirect Linear DNA, Invitrogen) to create recombinant baculovirus DNA. High-titre viral stocks were prepared and the titre was determined by end-point dilution [35]. The sequence of the final construct was verified by DNA sequencing.

Sf9 cells were grown in Sf-900 II SFM medium supplemented with 10% fetal bovine serum, 2% fungizone and 1% gentamicin at pH 6.2 in flasks for virus production, and in spinner flasks (stirred at 70 rev./min) or shaking bottles (at 135 rev./min) for protein expression. Cultures were maintained at 27 °C in a humid environment, and passaged every 7 days or when the density exceeded 2×10^6^ cells/ml. For protein expression, Sf9 cells were infected with ten virus particles/cell [MOI (multiplicity of infection)=10] and harvested after 60 h.

### Purification of IP_3_R1 from Sf9 cells

All procedures were performed at 4 °C, with incubations mixed by gentle rotation at 15 rev./min. Infected cells (3.2×10^9^) were harvested by centrifugation at 700 ***g*** for 5 min, washed with TEM (Tris/EDTA medium: 50 mM Tris/HCl, 1 mM EDTA, pH 8.3, and protease inhibitor cocktail) and centrifuged at 700 ***g*** for 5 min. The pellet was suspended in TEM, homogenized with an Ultra Turrax (9500 rev./min, two rounds of ten strokes) and then with a glass homogenizer (60 strokes), centrifuged at 900 ***g*** for 10 min, and the supernatant was then centrifuged at 46760 rev./min for 1 h using a Beckman 70.1 Ti rotor. The pellet was suspended in TEM (7 ml) and biotinylated by incubation for 12–16 h with D-biotin (100 μM) and biotin–protein ligase (BirA, 9 μg in a final volume of 10 ml) according to the manufacturer's instructions (Avidity). Membranes were recovered by centrifugation at 46760 rev./min for 1 h using a Beckman 70.1 Ti rotor.

Biotinylated membranes (16 mg of total protein/ml) were solubilized in PBM (phosphate-buffered medium: 100 mM Na_2_HPO_4_/NaH_2_PO_4_, 1 mM ETDA, 5% glycerol, 150 mM NaCl, pH 8, protease inhibitor cocktail and 1% DDM). The yield (~40%) after solubilization was lower than we and others have obtained using Triton X-100, but DDM is more widely used for solubilization of proteins for structural studies, and the background signals in EM were much lower with DDM than with Triton X-100. After 4 h, the supernatant was recovered after centrifugation at 46760 rev./min for 1 h using a Beckman 70.1 Ti rotor and incubated for 1 h with streptavidin–agarose beads (Invitrogen) at 2 mg of protein/100 μl of beads. The beads (100 μl) were washed twice with PBM and then resuspended in 100 μl of PrM (PreScission medium: 50 mM Tris/HCl, 150 mM NaCl, 1 mM EDTA, 1 mM dithiothreitol and 0.2% DDM, pH 7) containing PreScission protease (4 units/100 μl of beads). After incubation for 12 h, the supernatant was collected by centrifugation at 650 ***g*** for 5 min and applied to a HiTrap Q FF anion-exchange column, which was then washed (100 ml, 1 ml/min) with ion-exchange medium (50 mM Tris/HCl, 150 mM NaCl, 1 mM EDTA, 1 mM dithiothreitol, 5% glycerol and 0.02% DDM, pH 8.3). A linear gradient (150–500 mM NaCl in the same medium) was used to elute fractions of 0.5 ml, and the peak fractions (numbers 17–25) were pooled and concentrated to 0.5 ml with a Vivaspin-2 MWCO (molecular-mass cut-off) 30000 centrifugal concentrator (Sartorius). The concentrated sample was applied to a Superose 6 10/300 GL gel-filtration column, and fractions (0.5 ml) were eluted with ion-exchange medium (0.5 ml/min). Fractions 21–22 were pooled and used for EM.

Samples were analysed using pre-cast SDS/PAGE mini-gels (Invitrogen), and by immunoblotting using the iblot system (Invitrogen) with a rabbit peptide antiserum (1:1000) against the C-terminus of IP_3_R1 [12]. Anti-rabbit horseradish peroxidase-conjugated secondary antibody (AbCam, 1:5000) and Super Signal West Pico chemiluminescence reagent (Pierce) were used to detect immunoreactivity. The bands were quantified using GeneTools (Syngene). Protein gels were silver-stained using GelCode SilverSNAP II stain kit according to the manufacturer's instructions (Pierce).

### Ins(1,4,5)*P*_3_-evoked Ca^2+^ release from intracellular stores

The free [Ca^2+^] within the ER was recorded using a lowaffinity luminal Ca^2+^ indicator, Mag-fluo-4, and a FlexStation plate-reader (MDS Analytical Technologies) [34]. Sf9 cells (~2×10^6^/ml) were incubated for 1 h at 20 °C with Mag-fluo-4AM (20 μM) in Hepes-buffered medium (135 mM NaCl, 5.9 mM KCl, 11.6 mM Hepes, 1.5 mM CaCl_2_, 11.5 mM glucose and 1.2 mM MgCl_2_, pH 7.3) containing BSA (1 mg/ml) and Pluronic F127 (0.4 mg/ml). The cells were then resuspended in Ca^2+^-free CLM {cytosol-like medium: 20 mM NaCl, 140 mM KCl, 2 mM MgCl_2_, 1 mM EGTA, 375 μM CaCl_2_ (free [Ca^2+^] ~200 nM) and 20 mM Pipes, pH 7} containing saponin (10 μg/ml). After ~10 min at 37 °C, the permeabilized cells were washed (650 ***g***, 3 min), resuspended in Mg^2+^-free CLM with 10 μM FCCP (carbonyl cyanide *p*-trifluoromethoxyphenylhydrazone), distributed into 96-well plates (6×10^5^ or 2×10^5^ cells in 50 μl/well, for uninfected and infected cells respectively) and centrifuged at 1000 ***g*** for 3 min. After addition of MgATP (1.5 mM), the intracellular stores were loaded to steady-state with Ca^2+^, and, after 150 s, Ins(1,4,5)*P*_3_ was added with thapsigargin (1 μM); the latter to inhibit further Ca^2+^ uptake. Ins(1,4,5)*P*_3_-evoked Ca^2+^ release is expressed as a fraction of the ATP-dependent Ca^2+^ uptake. Concentration–effect relationships were fitted to a Hill equation using non-linear curve-fitting (GraphPad Prism, version 5).

### [^3^H]Ins(1,4,5)*P*_3_ binding

Equilibrium-competition binding assays were performed at 4 °C in TEM (500 μl) containing [^3^H]Ins(1,4,5)*P*_3_ (1.5 nM), membranes (~30 μg) or purified IP_3_R (~0.7 μg), and appropriate concentrations of Ins(1,4,5)*P*_3_. After 5 min, during which equilibrium was attained, incubations were terminated by the addition of ice-cold TEM (500 μl) containing PEG [poly(ethylene glycol)] 8000 (30%) and γ-globulin (20 μl of 25 mg/ml), mixed, incubated on ice for 5 min and then centrifuged at 20000 ***g*** for 5 min. The pellet was rinsed twice with 500 μl of TEM containing 15% PEG 8000, and then resuspended to allow its radioactivity to be determined by liquid scintillation counting. Results were fitted to a Hill equation using GraphPad Prism from which the IC_50_ and *K*_d_ were determined.

### EM and image analysis

Purified IP_3_R1 was loaded on to glow-discharged carbon-coated copper grids and negatively stained with 2% uranyl acetate. Micrographs were collected on a Tecnai T-12 electron microscope in low-dose mode (20 electrons/Å^2^) at a calibrated magnification of 41125, at 120 kV with ~700 nm defocus. The quality of the micrographs was assessed using an optical diffractometer. Only micrographs with circular and isotropic diffraction rings (Thon rings) consistent with a resolution of at least 20 Å within the first ring were used for further processing. Micrographs were digitized using a Nikon Coolscan 9000ED at a step size of 6.35 μm. Scanned micrographs were then converted for processing using IMAGIC programs [36] and 3-pixel×3-pixel areas were averaged, resulting in a final pixel size of 0.45 nm. The particles were selected with a box size of 100 pixels×100 pixels using the BOXER tool of the EMAN package [37]. IMAGIC [36] was used for all other image processing, except for the multi-reference alignment routine for which SPIDER [38] was used. In the first multi-reference alignment and first angular assignment, our previously published IP_3_R structure [23], filtered to 54 Å, was used as a reference. The resolution of the three-dimensional reconstruction was determined using the half-bit criterion.

## RESULTS AND DISCUSSION

### Expression, purification and characterization of recombinant IP_3_R1

After optimization of methods, the outcome of which is described in the Experimental section, we established that infection of Sf9 cells with the tagged IP_3_R1 construct shown in Figure 1(A) allowed the expression of functional IP_3_R at a level considerably exceeding that of endogenous IP_3_R. Immunoblotting confirmed expression of IP_3_R1 of appropriate size (~300 kDa, see below) in the infected cells. After permeabilization, control and infected cells were each able to accumulate Ca^2+^ after the addition of ATP. Ins(1,4,5)*P*_3_ caused a barely resolvable Ca^2+^ release from the control cells (maximal Ca^2+^ release, 7±1%; EC_50_, 0.50±0.05 μM; Hill coefficient, 0.9±0.17), but caused a very much larger release from the infected cells (maximal Ca^2+^ release, 47±2%; EC_50_, 1.6±0.41 μM; Hill coefficient, 1.1±0.17) (Figure 1B). In equilibrium-competition assays with [^3^H]Ins(1,4,5)*P*_3_ using membranes prepared from infected Sf9 cells, Ins(1,4,5)*P*_3_ bound with high affinity (*K*_d_, 10.49±1.06 nM, Hill coefficient, 0.80±0.08) and the density of the sites (*B*_max_) was 10.6±0.8 pmol/mg of protein (Figure 1C). The affinity of the tagged IP_3_R1 expressed in Sf9 cells is similar to that determined under the same conditions for native IP_3_R1 in cerebellar membranes [12] and of untagged IP_3_R1 expressed in Sf9 cells [23,39,40], and the level of expression is ~40-fold higher than that of native IP_3_R in Sf9 cells [40].

**Figure 2:**
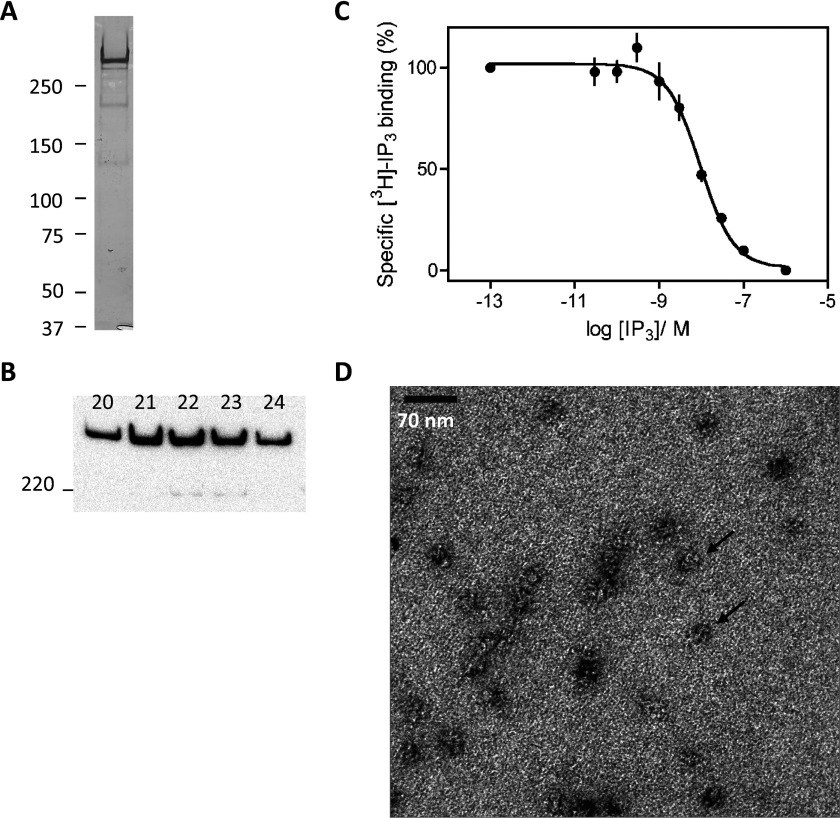
Purification of recombinant IP_3_R (**A** and **B**) Silver-stained gel (**A**, 0.12 μg of protein/lane) and immunoblot with an IP_3_R1-specific antiserum (**B**, 0.12 μg of protein/lane) of purified recombinant IP_3_R1 from fractions 20–24 of the gel-filtration step. Molecular-masses are shown in kDa. Results are typical of at least five similar analyses. (**C**) Specific binding of [^3^H]Ins(1,4,5)*P*_3_ (1.5 nM) in the presence of the indicated concentrations of Ins(1,4,5)*P*_3_ to purified recombinant IP_3_R1 (means±S.E.M, *n*=3). (**D**) Electron micrograph of purified recombinant IP_3_R1 highlighting particles (arrows) with the expected size of tetrameric IP_3_R (~20 nm diameter). Scale bar, 70 nm.

After biotinylation and solubilization (~40% yield), recombinant IP_3_R1 was purified using streptavidin and then cleavage by PreScission protease (~50% yield), anion-exchange (~16% yield) and size-exclusion (~70% yield) chromatography (see the Experimental section). Calibration of the final chromatography step suggested an oligomeric size for the purified IP_3_R of ~1.56 MDa, which is consistent with the predicted size of a tetramer of ECFP-tagged IP_3_R subunits (4×339 kDa) associated with DDM (see Supplementary Figure S1 at http://www.BiochemJ.org/bj/428/bj4280483add.htm). The final product migrated as a single band of appropriate size after SDS/PAGE and silver-staining (Figure 2A) or immunoblotting with an IP_3_R1-selective antiserum (Figure 2B). The *K*_d_ of the purified IP_3_R for Ins(1,4,5)*P*_3_ was 7.65±0.69 nM, the Hill coefficient was 1.1±0.09 (Figure 2C), and the final specific activity of the sites was 0.25±0.02 nmol/mg of protein (their concentration was 24 μg of IP_3_R/ml). Electron micrographs of purified IP_3_R1 showed particles of the expected size (~20 nm) with their 4-fold symmetry clearly apparent in several profiles (Figure 2D).

These results establish that we have successfully expressed functional IP_3_R1 at high levels in Sf9 cells and purified them under conditions that allow their structure to be analysed by EM and SPA.

### Three-dimensional structure of recombinant IP_3_R1

**Figure 3:**
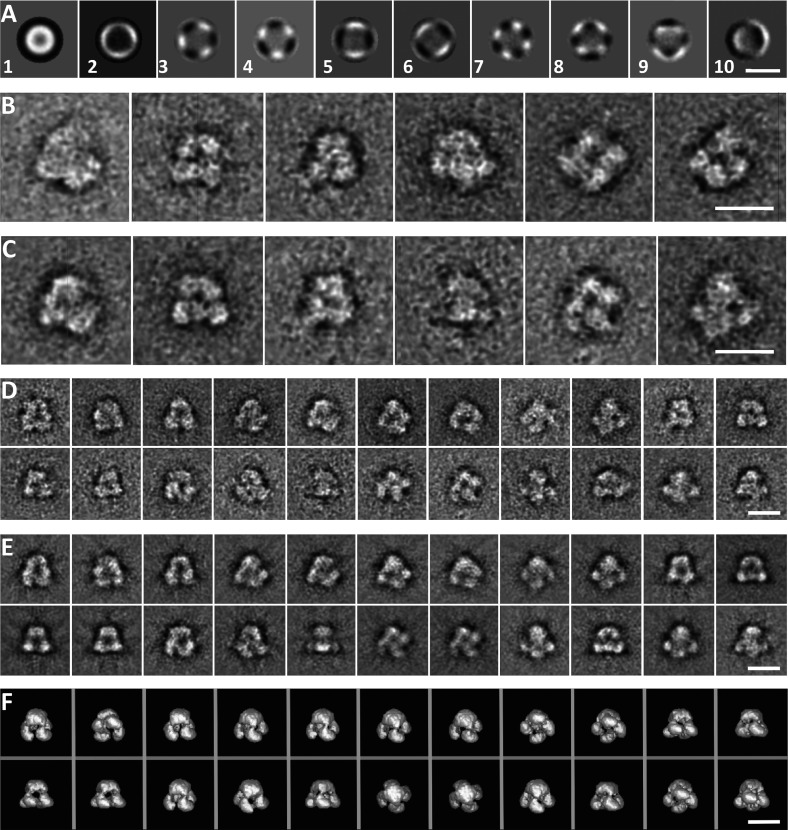
Image analysis of IP_3_R particles (**A**) Eigenimages of the unaligned dataset. Eigenimage 1 shows the sum of all images. Eigenimages 3 and 4 show approx. 3-fold symmetry. Eigenimages 5 and 6 show approx. 2-fold symmetry. Eigenimages 7 and 8 show 4-fold symmetry. (**B**) Class averages obtained independently after two iterative alignments (reference-free classes). (**C**) Similar class averages obtained after three iterative alignments using the filtered three-dimensional structure of native IP_3_R [23] as a reference for the first alignment. (**D**) Class averages used for the final three-dimensional reconstruction. (**E**) Re-projections of the final three-dimensional reconstruction corresponding to the class averages shown in (**D**). (**F**) Surface views of the final three-dimensional reconstruction in the same orientations as the class averages. Scale bars (**A**–**F**), 20 nm.

The final dataset included 7064 particles selected from scanned micrographs. Particles were picked only if they were separated from others and had a size appropriate for an IP_3_R (~20 nm). The centred, but otherwise unaligned, particles were subject to multivariate statistical analysis, which yielded 69 eigenimages; the first ten are shown in Figure 3(A). A 4-fold symmetry is clearly visible in eigenimages 7 and 8, and a 3-fold symmetry is visible in eigenimages 3 and 4. The apparent 3-fold symmetry may reflect a side view of the IP_3_R, which published structures have suggested may be approximately triangular [23,28]. Much of the data comprises these side views or near side views, with only a fraction of the unaligned data showing 4-fold symmetry. The 2-fold symmetry in eigenimages 5 and 6 is probably caused by particles that correspond to slightly tilted top or bottom views. Because the IP_3_R is tetrameric [26,41] (Supplementary Figure S1) and all previous EM analyses established a 4-fold symmetry [21–25], we applied C4 symmetry to the later stages of our three-dimensional reconstruction.

Class averages of the IP_3_R were calculated after reference-free alignment using IMAGIC and then refined using the SPIDER alignment routine using class averages of the initial reference-free alignment (Figure 3B). Figure 3(C) shows the class averages obtained using native IP_3_R [23] as a reference. For the three-dimensional reconstruction, recombinant IP_3_R particles were aligned using the filtered native IP_3_R structure [23] as a reference in the first iteration; these are similar to the reference-free class averages (Figure 3B). Well-preserved classes with good signal-to-noise ratios were selected and back-projected to produce three-dimensional maps used for further refinement of the classes. The class averages of each iteration cycle were used to obtain a three-dimensional reconstruction that was then used as a reference for the subsequent alignment. The three-dimensional reconstruction was stable after the third iteration. The fast stabilization of the analysis probably arises from our use of a refined structure of the IP_3_R [23] as an initial reference.

**Figure 4:**
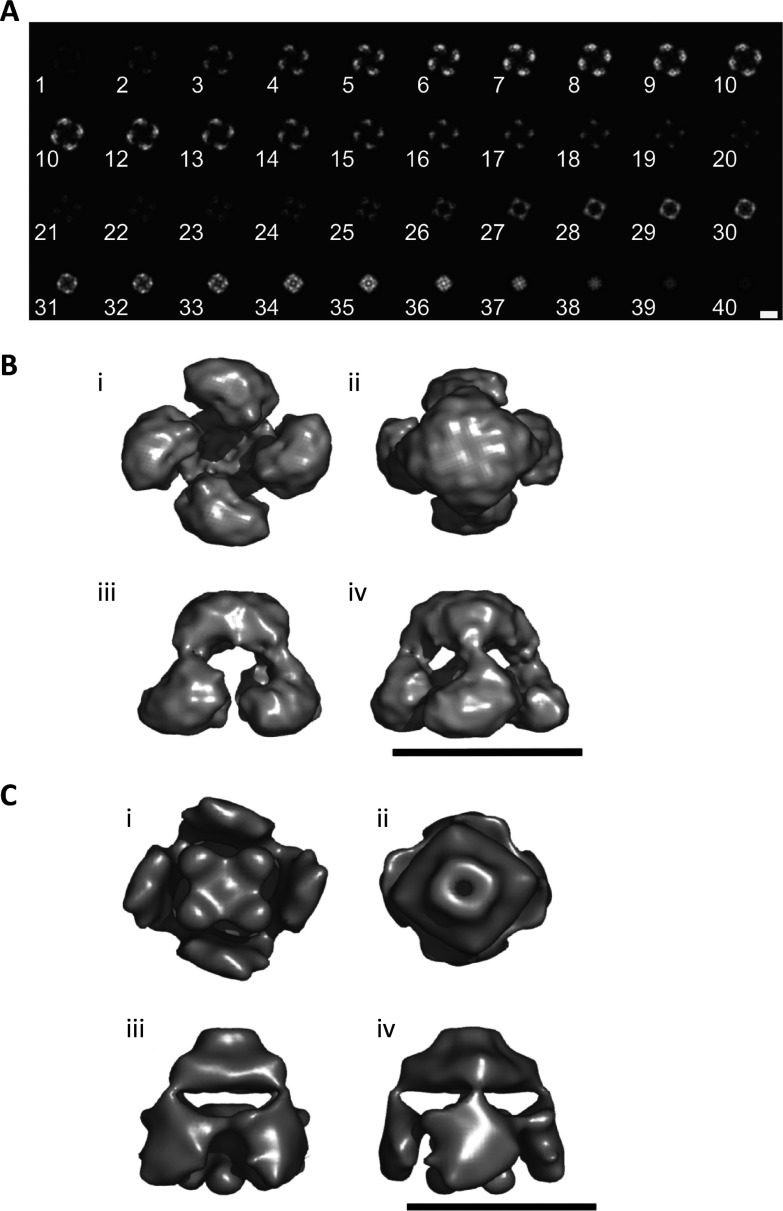
Structure of recombinant IP_3_R1 (**A**) Each section (1–40) is 0.45 nm thick and viewed along the symmetry axis starting from the cytoplasmic end (1) to the end likely to be within the ER lumen (40). Scale bar, 10 nm. (**B**) Surface views of the three-dimensional reconstruction of recombinant IP_3_R1 viewed from the cytosol (i), from the lumen of the ER (ii) and two views in cross-section (iii and iv). (**C**) Views similar to those shown in (**B**), but for native cerebellar IP_3_R [23]. Scale bars (**B** and **C**), 20 nm.

The final three-dimensional reconstruction contained 22 class averages with a good signal-to-noise ratio (Figures 3D–3F) and a wide distribution of Euler angles (see Supplementary Figure S2 at http://www.BiochemJ.org/bj/428/bj4280483add.htm). The resolution, measured using the half-bit criterion of the Fourier shell correlation, was approx. 40 Å (see Supplementary Figure S3 at http://www.BiochemJ.org/bj/428/bj4280483add.htm). The three-dimensional volume was contoured to accommodate a molecular mass of the recombinant IP_3_R1 of 1.3 MDa assuming a protein density of 844 Da/nm^3^. Figure 4(A) shows the structure of the recombinant IP_3_R as sections (each 0.45 nm thick) cut along the symmetry axis and Figure 4(B) shows four characteristic views of the final three-dimensional reconstruction. The overall dimensions of the three-dimensional structure are ~19.5 nm×19.5 nm×17.5 nm. It comprises a large cytoplasmic region (~19.5 nm×19.5 nm×10 nm) consisting of four petal-like domains (each ~10.6 nm wide) each connected to a smaller stalk-like channel region (~13.2 nm×13.2 nm×7.5 nm). This region tapers towards the luminal side to a width of ~7.4 nm (Figures 4A and 4B).

### Comparison of the structures of native and recombinant IP_3_R and RyR

The three-dimensional structure of recombinant IP_3_R1, which is likely to be in a closed state, resembles the flower model of the native IP_3_R1 [23] (Figure 4C), but without the central stigma. The overall dimensions of the two structures are also similar: ~18 nm×18 nm×18 nm for native IP_3_R1, and ~19.5 nm×19.5 nm×17.5 nm for recombinant IP_3_R1 (Figures 4B and 4C). The cytoplasmic region of each structure is ~11 nm high, and both have a putative channel region that tapers towards the luminal side to a width of ~7 nm. The only substantial difference between the two structures is the absence of a central stigma-like domain from the large cytoplasmic region of recombinant IP_3_R1 (compare Figures 4B, i and 4C, i). It is unlikely that this results from inappropriate assembly of the tetrameric IP_3_R because the recombinant protein was functional and bound Ins(1,4,5)*P*_3_ with appropriate affinity (Figures 1B and 1C). It may be that the N-terminal ECFP tag stabilized a different closed conformation of the IP_3_R or that the central stigma may represent an accessory protein [42] that is not present in the heterologous expression system. The three-dimensional structure of a native IP_3_R1 in a closed state from another group [21] and our structure of recombinant IP_3_R1 have similar overall dimensions (19 nm×19 nm×16 nm) and similarly sized channel domains (11 nm×11 nm×5.2 nm), and both lack a central stigma. However, the four cytoplasmic petal-like regions of our structure are discrete (Figure 4B, iii), but they are linked by slender bridges in the native structure [21]. A possible explanation is that the threshold set for the native model (1.7 MDa) is larger than the actual size of native IP_3_R (1.2 MDa), leading to inclusion of more density. The pinwheel structure of the native IP_3_R [24] has slightly larger dimensions (~25 nm×25 nm×19 nm), but it shares the four petal-like features of the recombinant IP_3_R structure, although their links with the channel region are both more substantial and more centrally placed than in our structure (Figure 4B). The two remaining structures of native IP_3_R [22,25] have similar dimensions to published structures of native IP_3_R and to our structure of recombinant IP_3_R1, but neither has obvious petal-like domains or a stalk-like channel domain.

RyRs and IP_3_Rs are relatives that share many structural and functional features, although IP_3_Rs are only half the size of RyRs. Both are cation channels with relatively weak selectivity for bivalent over univalent cations (*P*_Ba_/*P*_K_ ~7), although IP_3_Rs have lesser single-channel conductance than do RyRs [43,44]. The dimensions and tapering square profile of the channel region of RyR (~11.5 nm×11.5 nm×6 nm) [15] are similar to that of recombinant IP_3_R1 (13.2 nm×13.2 nm×7.5 nm) (Figure 4B). We note also that the large cytoplasmic region of RyR, like that of recombinant IP_3_R1, has a large central cavity rather than a stigma (Figure 4B).

In summary, we have provided the first three-dimensional structure of a recombinant IP_3_R at a resolution of ~40 Å. The dimensions of our structure and its essential features are similar to the shared structural features of IP_3_R purified from native sources (Figure 4). This establishes the utility of recombinant IP_3_R and EM and SPAs for further elaboration of the structural determinants of IP_3_R behaviour.

## Online data

Supplementary data
