# Supplementary material for: Three-dimensional structure of recombinant type 1 inositol 1,4,5-trisphosphate receptor
Source: Biochem J. 2010 May 27;428(Pt 3):483–9. doi: 10.1042/BJ20100143 (PMC3685215; doi:10.1042/BJ20100143)
Supplement: Supplementary data [file bj4280483add.pdf]

BJ www.biochemj.org

Biochem. J. (2010) **428**, 483–489 (Printed in Great Britain) doi:10.1042/BJ20100143

SUPPLEMENTARY ONLINE DATA

Three-dimensional structure of recombinant type 1 inositol 1,4,5-trisphosphate receptor

Francis WOLFRAM*, Edward MORRIS† and Colin W. TAYLOR*[1]

*Department of Pharmacology, University of Cambridge, Tennis Court Road, Cambridge CB2 1PD, U.K., and †Section of Structural Biology, Institute of Cancer Research, Chester Beatty Laboratories, London SW3 6JB, U.K.

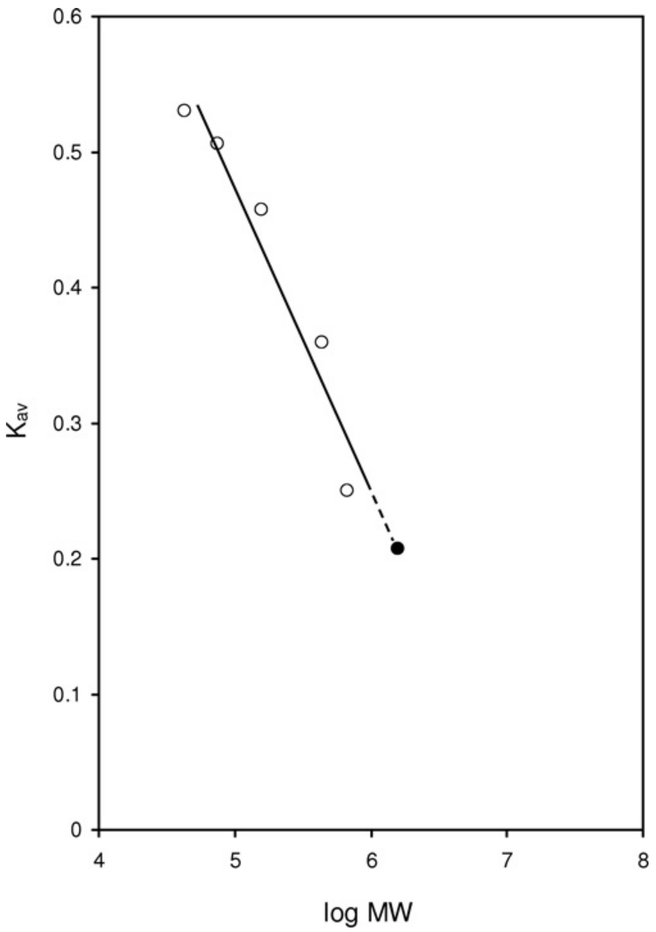

Figure S1 Estimated size of purified IP_3R1 from gel filtration

Calibration of the Superose 6 10/300 GL column used for the final purification step allowed the molecular mass of the purified IP_3R1 to be estimated. The standards used were: ovalbumin, 43 kDa; conalbumin, 75 kDa; aldolase, 158 kDa; ferritin, 440 kDa; and thyroglobulin, 669 kDa (gel filtration calibration kit HMW, GE Healthcare, ○). The gel-phase distribution coefficient (K_{av}) was calculated from [1]: $K_{av} = (V_e - V_0)/(V_c - V_0)$ where V_e is elution volume, V_0 is void volume (determined using Blue Dextran), and V_c is column volume. From the calibration curve [K_{av} against log molecular mass (MW), continuous line], K_{av} for the purified IP_3R1 suggests a molecular mass of 1.56 MDa (●). Under the conditions used for IP_3R1 purification (0.02% DDM), the DDM micelle (aggregation number >140 for concentrations above the critical micellar concentration) is likely to contribute a further molecular mass of at least 70 kDa. We conclude that the observed mass (1.56 MDa) is consistent with the purified recombinant IP_3R1 being a tetramer (4×339 kDa = 1.36 MDa) associated with DDM and perhaps also endogenous lipids.

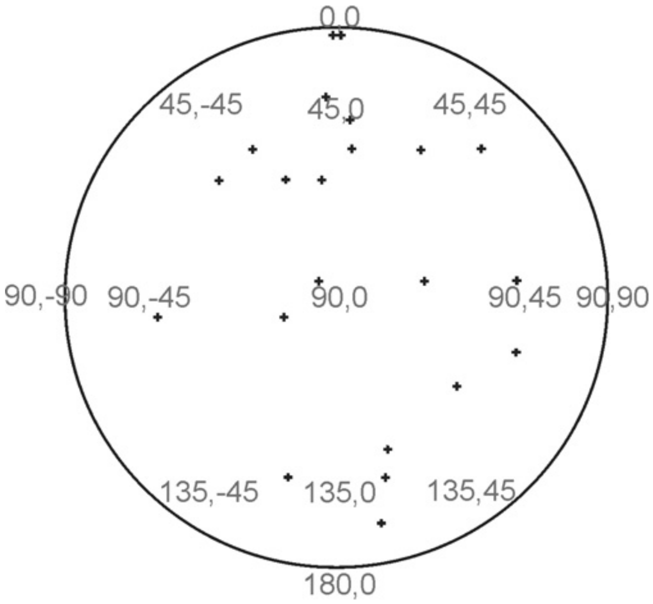

Figure S2 Euler angle distribution of final class averages

The Euler angle sphere shows the assigned Euler angles of the 22 class averages used to obtain the final three-dimensional reconstruction. Each class average image is assigned three Euler angles to describe the rotations that have to be performed to obtain the desired view of the three-dimensional structure. The α angles are set to 0° and are not shown; the other two angles define where the images are within the Euler sphere.

[1] To whom correspondence should be addressed (email cwt1000@cam.ac.uk).

© 2013 The Author(s)
The author(s) has paid for this article to be freely available under the terms of the Creative Commons Attribution Licence (CC-BY) (http://creativecommons.org/licenses/by/3.0/) which permits unrestricted use, distribution and reproduction in any medium, provided the original work is properly cited.

Biochemical Journal

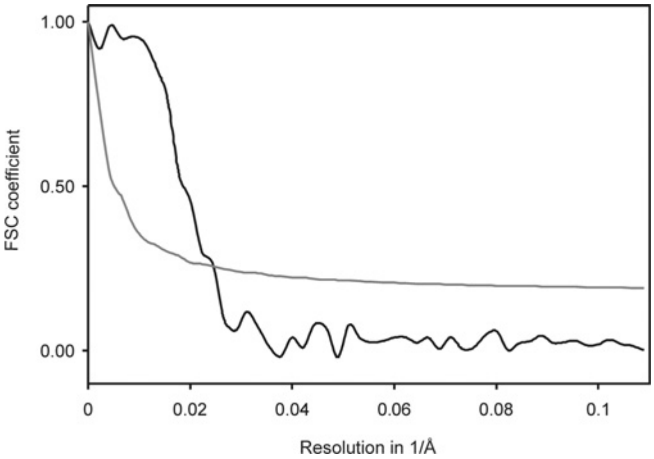

Figure S3 Resolution of the final three-dimensional reconstruction

The Fourier shell correlation (FSC) coefficients were calculated for the two subsets of the final class averages and plotted (black line) together with the half-bit curve (grey line). The resolution of the final three-dimensional reconstruction, given by the crossing of the two curves, is ∼40 Å.

REFERENCE

1 Wilson, K. and Walker, J. (2000) Principles and Techniques of Practical Biochemistry, 5th., Cambridge University Press, Cambridge

Received 25 January 2010/29 March 2010; accepted 8 April 2010
Published as BJ Immediate Publication 8 April 2010, doi:10.1042/BJ20100143

© 2013 The Author(s)
The author(s) has paid for this article to be freely available under the terms of the Creative Commons Attribution Licence (CC-BY) (http://creativecommons.org/licenses/by/3.0/) which permits unrestricted use, distribution and reproduction in any medium, provided the original work is properly cited.
